# Clinical Profile and Social Cognition in Patients with Psychotic Symptoms Related to DiGeorge Syndrome: A Case-Control Study

**DOI:** 10.1192/j.eurpsy.2026.12176

**Published:** 2026-07-31

**Authors:** I. Berardelli, A. Comparelli, S. Sarubbi, C. Berni, M. D’orsi, F. Formica, R. Iannazzo, F. Schirripa, M. Pompili

**Affiliations:** 1 Department of Neurosciences, Mental Health and Sensory Organs, Faculty of Medicine and Psychology, Suicide Prevention Centre, Sant’Andrea Hospital, Sapienza University of Rome, Via di Grottarossa, 1035, 00189, Rome, Italy; 2 Psychiatry Residency Training Program, Faculty of Medicine and Psychology, Sapienza University of Rome, Psychiatry Unit, Sant’Andrea Hospital,00185, Rome, Italy, Rome, Italy

## Abstract

**Introduction:**

The 22q11.2 deletion syndrome (22q11DS) is a genetic condition representing a model of vulnerability to psychotic disorders (schizophrenia and schizoaffective-like) and mood disorders. Affected individuals show also cognitive and social impairments that impact global functioning and quality of life.

**Objectives:**

This case-control study examined the associations between psychopathological and cognitive domains in 22q11DS patients compared with non-syndromic schizophrenia, aiming to clarify how genetic vulnerability, cognitive functioning and social cognition (SC) interact to constitute an endophenotype of psychotic risk.

**Methods:**

Forty-two patients with schizophrenia spectrum disorders were included: 21 with 22q11DS and 21 with non-syndromic schizophrenia, matched for sex and diagnosis. Cognitive domains were assessed with the Cognitive Assessment Interview (CAI), and psychotic symptoms were evaluated using the Positive and Negative Syndrome Scale (PANSS).

**Results:**

Patients with idiopathic schizophrenia showed significantly higher severity of positive, general, cognitive, excitement, and depression symptoms (all p < .05). By contrast, patients with 22q11.2DS exhibited more pronounced SC impairment (U = 81.00, p < .001). In 22q11.2DS, SC correlated with multiple cognitive and clinical domains, including working memory, attention/vigilance, processing speed, reasoning/problem solving, and PANSS total score. Correlation analyses revealed significantly stronger links between negative symptoms and cognitive domains in 22q11.2DS compared to schizophrenia. Regression analysis identified verbal learning/memory as the only independent predictor of SC (β = .56, p = .010), explaining 86% of variance (R² = .86).

**Conclusions:**

This case-control study suggests that in 22q11DS, genetic vulnerability translates into basic cognitive deficits that contribute to social-cognitive and functional impairment. The profile, partially distinct from idiopathic schizophrenia, emphasizes that social cognition should be a central target for clinical and rehabilitative interventions.

**Disclosure of Interest:**

None Declared

